# Agent-Based Simulation of Virus Testing in Certain-Exposure Time through Community Health Service Centers’ Evaluation—A Case Study of Wuhan

**DOI:** 10.3390/healthcare9111519

**Published:** 2021-11-08

**Authors:** Xingyu Zhou, Jie Zhao, Duanya Zheng, Yang Yu, Lingbo Liu

**Affiliations:** 1 Department of Urban Planning, Wuhan Planning & Design Institute, Wuhan 430014, China; zxy1987913@sina.com; 2 Department of Urban Planning, School of Urban Design, Wuhan University, Wuhan 430072, China; yuyang1@whu.edu.cn (Y.Y.); lingbo.liu@whu.edu.cn (L.L.); 3 Wuhan Institute of Landscape Architectural Design Co., LTD, Wuhan 430025, China; zdy19860901@sina.com

**Keywords:** certain-exposure time, agent-based traffic simulation, community health service facilities, layout evaluation

## Abstract

Short-term and large-scale full-population virus testing is crucial in containing the spread of the COVID-19 pandemic in China. However, the uneven distribution of health service facilities in terms of space and size may lead to prolonged crowding during testing, thus increasing the chance of virus cross-infection. Therefore, appropriate control of crowd exposure time in large-scale virus testing should be an important goal in the layout of urban community health facilities. This paper uses the Quanta concept and Wells-Riley model to define the “certain-exposure time” under low cross-infection rate. Then, an agent-based simulation model was used to simulate the reasonable screening efficiency of community health service facilities during certain-exposure time at different stages of the COVID-19 pandemic and under different screening processes. Eventually, the screening efficiency was evaluated for all community health service centers in Wuhan. During the early period of the pandemic, 23.13% of communities failed to complete virus testing of community residents within 2 h of certain-exposure time, leaving approximately 56.07% of the population unscreened; during the later period of the COVID-19 pandemic, approximately 53% of communities and 75% of residents could not be screened. The results can pinpoint the distribution of community health service centers with inadequate screening capacity, facilitate targeted policymaking and planning, and effectively curb COVID-19 cross-infection during screening.

## 1. Introduction

The COVID-19 pandemic has swept through the world and caused great casualties and economic losses. China’s experience in combating the epidemic has demonstrated that mass virus testing can effectively detect infected cases and potential transmitters and assess the overall situation of pandemic prevention and control in urban areas [1,2,3]. Residential communities are basic units of large-scale virus testing, but community health services-based virus testing faces the challenges of large population, heavy workloads, and tight schedules. Large-scale and prolonged queues of residents for testing are likely to increase the risk of virus infection among residents [4,5,6]. Therefore, a reasonable layout of community health service facilities and an efficient testing process have a significant impact on reducing the infection rate of respiratory infectious diseases under major epidemics.

By combining and correlating the findings of several studies, it can be concluded that reducing the exposure time of people in public places (e.g., hospitals) that may contain infected persons can significantly reduce the chance of infection, with respiratory infectious diseases including COVID-19 [7,8]. In general, studies suggest that more than 7.5% of patients in hospitals are cross-infected. In the COVID-19 pandemic period, a study concluded that 41% of patients in Wuhan were infected by hospital visits, and the prolonged exposure time to infected patients may be the main reason of infection [9,10]. Qian-Hua once counted the SARAS outbreak records in several hospitals in Taipei and Hong Kong and found that, altogether, patients were with infected persons for at least 2 h of exposure time [11]. Many medical experts suggest that the contact time of health care workers with SARAS patients in a single visit should not exceed 2 h [12]. Based on this, further quantitative studies on the association between exposure time and risk of infection were conducted by scholars. Liao Yue used the Wells-Riley model to simulate the indoor spatial transmission of respiratory infectious diseases (some parameters were obtained by CFD fluid modeling) and found that the chance of infection increased geometrically with increasing exposure time [13].

The exposure time of people in public places can be reduced by a reasonable organization of clinical flow. In this field, early studies used queuing theory to calculate the service efficiency of clinical flow, but this theory ignored the influence of pedestrians, space and other realistic elements on clinical flow, and it was difficult to provide effective suggestions at the facility layout level. Later, with the development of traffic simulation modeling technology, models such as cellular automata (CA) were introduced to simulate the operation of clinical flow in real space [14,15]. However, the CA model emphasizes that each cell must follow the same principles and rules [16], which is different from the actual situation in which individuals have complex multi-branch decisions in the clinical flow, and the final simulation results tend to deviate from the actual situation. In response to this phenomenon, some scholars have proposed the introduction of an agent-based model to optimize the simulation [17,18,19]. The agent model emphasizes the autonomy of individuals and the suitability of individuals to modify their behavior to adapt to the changes in the external environment, which allows the agent model to more clearly reflect the different decision-making states of individuals in the clinic flow and the resultant series of interactions, which are more closely related to the actual situation. With the agent model, some scholars have carried out the COVID-19 transmission simulation at a macro level [20]. However, more scholars use the agent model to study the spread and prevention and control of the COVID-19 in hospitals, schools, supermarkets, and other large public spaces by virtue of its advantages in micro-scale simulation [21,22,23,24,25,26]. In the most exciting research [27], the blood collection and diagnostic flow system in hospitals were optimized by using this system. During the period of COVID-19, there was research focused on the epidemic prevention and control in school public space and the operation of logistics system transfer by this software [28,29]. However, there is relatively little research on community-level health service facilities. Most of the evaluation studies conducted at the level of community health service facilities are aimed at improving the capacity of basic disease services, preferring to expand the scale of community health service facilities, increase the distribution of community health service facilities, or improve the satisfaction users. However, there are insufficient targeted studies or real-world simulations on the efficiency of viral testing in community health service centers during the COVID-19 pandemic.

**Figure 1:**
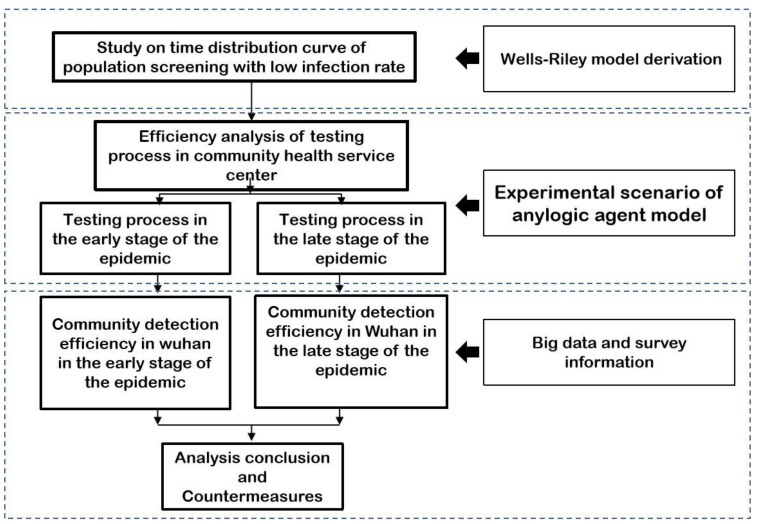
The technical route to construct and analyze the virus testing of COVID-19.

Thus, most of the studies have been conducted in large public spaces and there is a lack of evaluation studies on community health service facilities. They have failed to incorporate reasonable exposure-time settings for virus testing into clinic flow simulations and they cannot ensure low cross-infection at the large-scale virus testing stage. Therefore, in this paper, based on community population data and community health service centers data, we simulated the testing process in community health service centers as a microscopic scenario based on Wells-Riley and agent-based simulation models to obtain the maximum virus testing efficiency in the community within certain-exposure time. Then, we evaluated the community health service facilities throughout Wuhan City to identify the weak areas at the community virus-testing stage (Figure 1) in order to provide more accurate and better medical conditions for in-depth disease control and prevention, reduce the chance of infection, and provide technical support and assistance for the organization of possible large-scale virus testing in the future.

According to the technical route, this paper will analyze the changes in virus transmission probability in a certain time according to the wells Riley formula and CFD fluid model and complete the multi simulation scenario construction with Anylogic according to the layout of community medical facilities in different period, different virus detection process and detection population size. Finally, relying on the distribution of community residents in Wuhan, the status of virus monitoring facilities in community medical and health centers, complete the simulation evaluation of the actual virus detection in Wuhan, and put forward corresponding suggestions.

## 2. Data and Methodology

### 2.1. Data Collection and Processing

Two types of data were used in this paper. One is the demographic and spatial data, including the size of the community, the size of the community residents, the size and spatial distribution of community health service centers, and the behavioral characteristics of the residents for virus testing. The other category is medical data, including the process of virus testing before and after the COVID-19 pandemic, the availability of healthcare personnel and health service facilities in community health service centers (Table 1).

At the beginning of the COVID-19 pandemic, Wuhan city re-collected the population size data in the form of the grid (500 m × 500 m), which shows that the city has a population of about 10.58 million. There are 183 communities in Wuhan, including 80 in the main city within the Third Ring Road and 103 outside the Third Ring Road. There are 204 community health service centers, ensuring that each community has at least one health service center, and there are 179 community health service stations. Community health service centers are larger and better equipped to detect viruses in the early and late stages of COVID-19 infection, while health service stations can only detect viruses in the later stage of COVID-19 infection. In this paper, we obtained the data of community health service centers and health service stations with high, medium, and low population densities through field survey, and averaged the data of medical and nursing staffs and equipment configuration of both. Finally, we collected the help-seeking data from Sina Weibo and the data published in the official news and set the proportions of the population in need of initial and second temperature measurement, throat swab test, blood test and CT scan among the whole population tested.

### 2.2. Simulation of the Size of Residents Tested by Community Health Service Centers in Different Periods

Before April 2020, due to the lack of understanding about COVID-19, being infected or not was confirmed through not only throat swabs and blood drawing, but also CT film. Meanwhile, a large number of residents had a sense of panic, which made them flock into the hospital for checking whether infected or not, resulting the short of medical resources. Therefore, multiple rounds of temperature detection were needed to pass a number of “hysteria” residents who were not infected with the virus in order to prevent wasting precious medical resources. We define this panic and confusion moment as the early period. After April 2020, with the improvement in the detection technology, the detecting process was greatly simplified, and only throat swabs and blood draws were needed, thus reducing the panic among the residents and we define this as the late period. As the division of the two periods shows, the cognition, detection, prevention, and control of respiratory epidemics, including COVID-19, are not unchanged but gradually progressive. At the same time, the corresponding research simulation and countermeasure suggestions also need to keep pace with the times.

According to a survey by the voting of over 400,000 users on Sina Weibo at the early period of the COVID-19 pandemic [30], about 41.53% of the voters suspected that they were infected with COVID-19. Therefore, the same proportion of residents at the beginning of the COVID-19 pandemic is set to be tested at their community health service centers according to the “graded diagnosis ”. Another survey on Weibo showed that 90% of residents in the initial phase of the COVID-19 pandemic had a normal initial temperature, so only 10% of residents were set to have a second temperature measurement.

In addition, referring to the proportion of residents and febrile residents seen by the community health service centers in Wuhan on 8 February 2020 [31], 23.12% of residents required further blood tests and CT scans after the second temperature measurement.

### 2.3. Methodology

#### 2.3.1. Quanta Concept and Wells-Riley Model

Respiratory infectious diseases are transmitted by airborne droplets containing pathogens through breathing, talking, coughing, and sneezing. The probability of infection is related to a number of factors that are difficult to precisely determine, such as the type and number of pathogens, host susceptibility, and the external environment. Wells proposed the concept of “Quanta” in 1995 to determine the probability of infection of airborne infectious diseases [32]. According to Quanta’s definition, a patient needs to inhale a certain number of pathogens to become infected, and the average infection rate of inhaling 1 Quanta dose should follow the Poisson distribution, which means that there is 63.2% (1 − e^−1^) probability of contracting respiratory infectious diseases. Riley proposed the Wells-Riley model based on this theory combined with the prediction Formulas of classical probabilistic models of infectious diseases [33,34,35]. Later, Seppanen, Fennelly, Rudnick, Nicas et al., continuously improved and optimized the model to make the calculation of Quanta more scientific. Here is an example of Nicas’ improved Wells-Riley formula (Formula (1)).

(1) $$R\left(z,t_{l}\right)\approx 1-\exp\left(\sum_{j=1}^{m}r_{j}\beta_{j}c_{0}p\sum_{k=1}^{1}\sum_{i=1}^{n}c{\left(x_{j},t_{k}\right)}_{j}f\left(t_{r}\right)\Delta t_{k}\right)$$

where *R*(*z,t_l_*) is the risk of infection in the respiratory zone at exposure time *t_l_*, m is the total number of droplet particles in the range, n is the number of droplet particles in the respiratory zone, *r* is the fitted infection parameter, *β* is the respiratory deposition fraction, *c*_0_ is the initial concentration of virus in the droplet, p is the lung ventilation of the Subject, ƒ(*t_r_*) = pathogen survival Formula, Δ*t_k_* = *t_k_* − *t_k−_*_1_, *t_k_* = 0.

At the same time, the CFD fluid model can be used to simulate the parameters of the modified Wells-Riley model, and the Quanta values can be directly calculated (instead of relying on observed statistics).

#### 2.3.2. Agent-Based Simulation Model

An agent is a socially interactive and intelligent individual, which is mostly used in the field of computer and artificial intelligence [36,37,38]. In the field of agent simulation, the agent model is used to give the intelligent and social personality of real pedestrians to the virtual pedestrians in the simulation model, which effectively improves the scientificity of pedestrian traffic simulation at microscopic scale [39,40,41].

Anylogic is one of the most common software for simulating agent models in current research. The advantage of its agent modeling and simulation technology is the introduction of social force model to explain the behavioral characteristics of pedestrians [42]. The social force model proposes that pedestrians’ actions are influenced by three forces: first, their own drive, i.e., their desire for a destination; second, the forces between pedestrians, including psychosocial forces that decrease with distance and physical forces that occur with contact; and third, the forces between pedestrians and obstacles, similar to the second force, including psychosocial and physical forces of pedestrians after encountering obstacles. The model is expressed by the following formula [43]:

(2) r→αddt=v→αt

(3) v→αddt=f→αt+ε→αt

(4) $${\vec{f}}_{\alpha }\left(t\right)={\vec{f}}_{\alpha }^{ο}\left({\vec{v}}_{\alpha }\right)+{\vec{f}}_{\alpha \beta }\left({\vec{r}}_{\alpha }\right)+\sum_{\beta \left(\neq \alpha \right)}{\vec{f}}_{\alpha \beta }\left({\vec{r}}_{\alpha },{\vec{v}}_{\alpha },{\vec{r}}_{\beta },{\vec{v}}_{\beta }\right)+\sum_{i}{\vec{f}}_{\alpha i}\left({\vec{r}}_{\alpha },{\vec{r}}_{j},t\right)$$

Formula (2) represents the kinetic Formula, ${\vec{r}}_{\alpha }$ denotes the spatial vector of pedestrian *α*, and ${\vec{v}}_{\alpha }$ denotes the velocity of pedestrian *α*. Formula (3) represents the Formula of pedestrian acceleration and deceleration and direction change, ${\vec{f}}_{\alpha }\left(t\right)$ denotes the social force and ${\vec{\varepsilon }}_{\alpha }\left(t\right)$ denotes perturbation term of random behavior deviation; Formula (4) represents the Formula of social force, ${\vec{f}}_{\alpha }^{ο}\left({\vec{v}}_{\alpha }\right)$ denotes the acceleration force, ${\vec{f}}_{\alpha \beta }\left({\vec{r}}_{\alpha }\right)$ denotes the force between human and obstacle,${\vec{f}}_{\alpha \beta }\left({\vec{r}}_{\alpha },{\vec{v}}_{\alpha },{\vec{r}}_{\beta },{\vec{v}}_{\beta }\right)$ denotes the force between pedestrian *α* and other pedestrians, and ${\vec{f}}_{\alpha i}\left({\vec{r}}_{\alpha },{\vec{r}}_{j},t\right)$ denotes the attraction effect.

The agent model can realistically simulate the self-organized behaviors of pedestrians during travel, such as queuing, short cutting, grouping and other anthropomorphic choices and operations, thus portraying the complex behaviors of groups. It provides a more scientific perspective for the spatial simulation and quantification of group pedestrian flow, which can more accurately simulate and optimize the flow in medical facilities in a limited space scenario.

#### 2.3.3. Prerequisite Setup for Simulation Run

**Figure 2:**
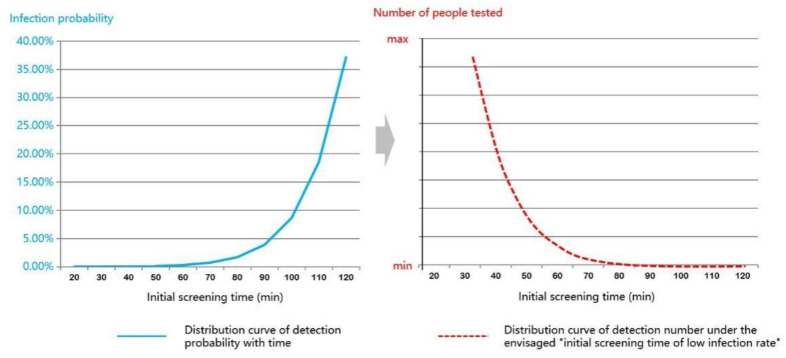
Distribution of infection rate and times of patients in virus testing time of 20–120 min.

When using Anylogic to simulate the clinic flow for virus testing, we should set the operating premise based on the safety of testing. In this paper, we simulated the airborne parameters of various influenza viruses in Formula (1) based on the Wells-Riley model and combined with the CFD fluid model [44,45], assuming a consistent airborne concentration of viruses inside community health service centers to simulate the probability of virus transmission within 120 min (Figure 2).

The figure shows a “monotonically increasing convex function curve” between the testing exposure time and the probability of infection (solid blue line in Figure 2). As the exposure time increases, the infection rate of the population increases exponentially. Therefore, from a statistical point of view, the inversion of the function curve suggests that when the testing efficacy results in a decreasing number of people tested within 2 h of exposure equivalent to the simulated curve, the probability of virus infection in the population is minimized. Therefore, in the agent simulation model, the testing exposure time was set to two hours, and the testing efficacy simulation should be consistent with the simulation curve to the maximum extent.

#### 2.3.4. Simulation Rules Setup

The COVID-19 pandemic was divided into the early and the late periods based on the changes in the testing process. The changes in the testing process resulted in a large change in the testing tasks undertaken by community health service centers. In this paper, we simulate the testing efficiency of community health service centers in different periods (Table 2).

1. Testing process during the early period of the COVID-19 pandemic

**Figure 3:**
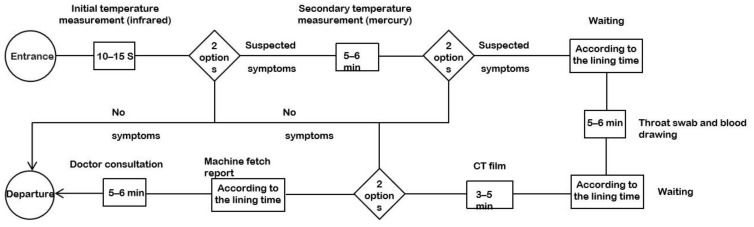
Community virus-testing workflow.

At the beginning of the COVID-19 pandemic, residents went to all levels of hospitals for testing in large numbers due to panic. Due to the lack of knowledge about the virus, the testing process included temperature testing, throat swab, blood sampling, and CT scan. As required by the city government, residents were required to take the initial temperature test (infrared thermometer) at the community health service centers. Patients with suspected fever were given a second temperature test (mercury thermometer). After the patient’s fever was confirmed, the patient was asked to undergo throat swab testing, blood sampling, CT scan and physician consultation for reporting (the final decision of whether to confirm the diagnosis was made by the higher-level fever clinic). The testing process was interspersed with residents moving, waiting in line, and talking, forming different branching options (Figure 3).

**Figure 4:**
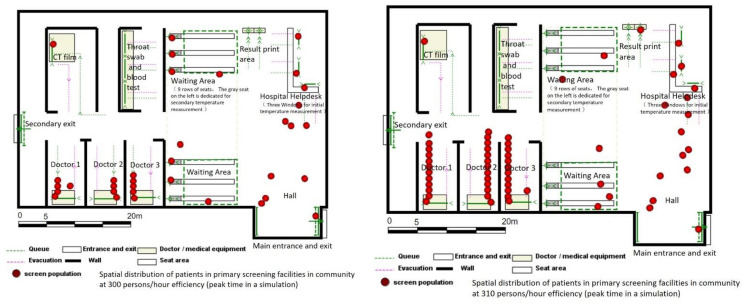
Schematic diagram of community virus testing facilities spatial distribution planning.

According to published data from Wuhan [16] and field visits, community health service centers need to isolate an indoor space of about 700 square meters for testing in order to implement the above testing process. In addition, a corresponding number of testing facilities should be equipped to form the spatial layout of virus testing in community health service centers during the initial phase of the COVID-19 pandemic (Figure 4).

At the same time, based on the community testing management method by notifying virus testing for different buildings at different times, we can ensure that the number of residents participating in hourly testing is basically steady and continuous, forming a “Poisson flow”.

2. Testing process in the late COVID-19 pandemic

**Figure 5:**
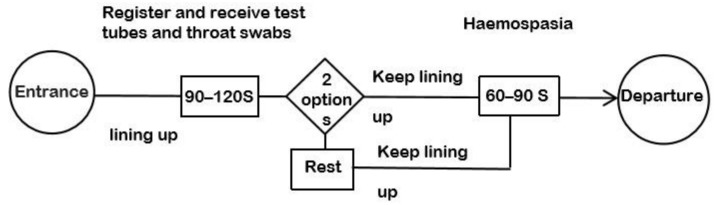
Community virus-testing workflow in the late COVID-19 pandemic period.

With the advancement of virus-testing technology, the process of CT scan and doctor consultation was eliminated during the late COVID-19 pandemic period, and the testing process was streamlined to two steps: registration for a test tube, throat swab, and blood sampling. At this stage, virus testing was opened to all citizens, and the scale of testing increased significantly (Figure 5).

**Figure 6:**
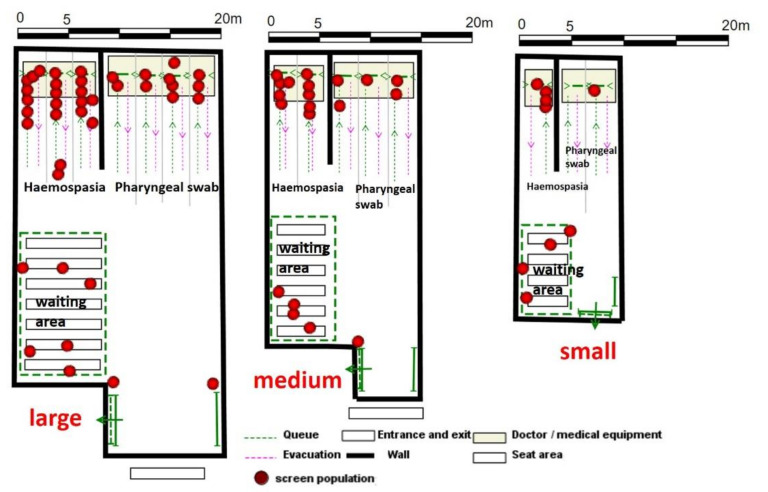
Schematic diagram of Community virus testing facilities spatial distribution planning.

The layout of the testing space at this stage is simpler and more diverse. Field survey revealed that the space can be divided into three categories according to its size: 250 m^2^, 450 m^2^ and 700 m^2^ (Figure 6).

Among them, the throat swab testing windows were set at 4 for large spaces, 3 for blood sampling, 3 and 2 for medium-sized spaces, and 2 and 1 for small spaces, respectively. According to the survey and the first list of community nucleic acid monitoring institutions disclosed by the municipal government, 21 community health service centers in Wuhan currently adopt large-space standards, while other community health service centers generally adopt medium-space standards and health service stations mainly adopt small-space standards.

## 3. Results

### 3.1. Efficiency Analysis of Standardized Community Testing Services Based on Certain-Exposure Time

Based on the certain-exposure time, we used the agent model of Anylogic to build various experimental scenarios of “spatial model of testing rooms in community health service centers” to analyze the testing efficiency of community health service centers under different conditions, based on the testing process and characteristics of different periods of the COVID-19 pandemic.

#### 3.1.1. The Early COVID-19 Pandemic Period

Based on the testing process and voting data of the early COVID-19 pandemic period, it can be inferred that about 101,700 people out of 10.58 million residents in Wuhan during the COVID-19 pandemic had a complete set of tests, from 2 temperature measurements to throat swab, blood sampling to CT scan (representing suspected symptoms). This size setting is deemed accurate when compared to the final number of more than 50,000 confirmed COVID-19 cases in Wuhan (the number of confirmed cases represent about 50% of the number of residents tested).

**Figure 7:**
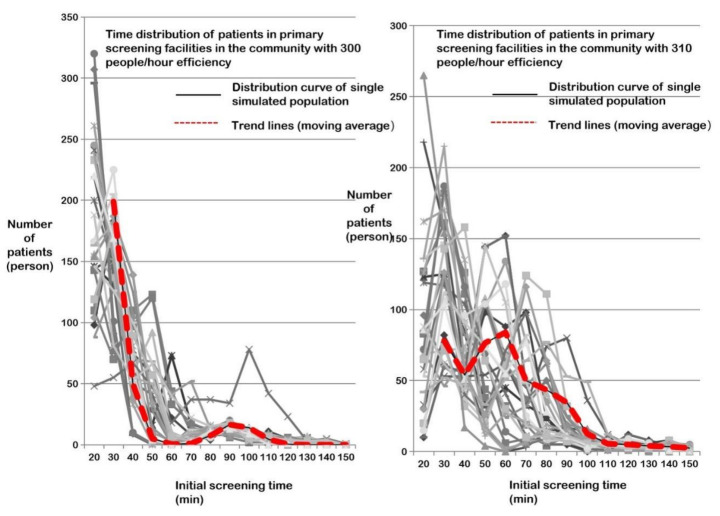
Statistical chart of residents’ time distribution in community primary testing at the efficiency of 300–310 persons/h (30 times for each simulation).

After the conditions were set, the simulation commenced. Considering the uncertainty of the length of each step in the clinic flow and the subsequent step of the Subject’s behavior, the simulation was repeated 30 times for each assumed service efficiency (the number of Subjects placed in the queue for testing per unit time), and the average service efficiency of community health service centers (i.e., the distribution of testing time spent by all Subjects) was checked to see if it coincided with the curve. In the simulation, it was found that the number of Subjects had a high impact on the testing efficiency, and an increase in only 10 persons/h in a fixed spatial scale and experimental scenario would lead to an exponential increase in the aggregation of residents to be tested, and a significant increase in the group testing time (Figure 7). After debugging, it was determined that when the maximum equivalent number of people to be tested is controlled within 300 persons/h, the residents can basically complete the testing within 2 h. In the simulation results, only 93 people (i.e., the average value of 3 people per simulation) were tested in more than 2 h, and the excess time could be controlled within 10 min. The overall fitted trend line (using the moving average) is very close to the previously expected “certain-exposure time” infection distribution curve (blue curve in Figure 2), indicating a relatively ideal service efficiency of the testing facilities.

This efficiency is about 15–20% lower than the maximum service efficiency of community testing facilities, but this reduction in efficiency is acceptable to achieve testing within a certain-exposure time.

#### 3.1.2. The Late COVID-19 Pandemic Period

**Figure 8:**
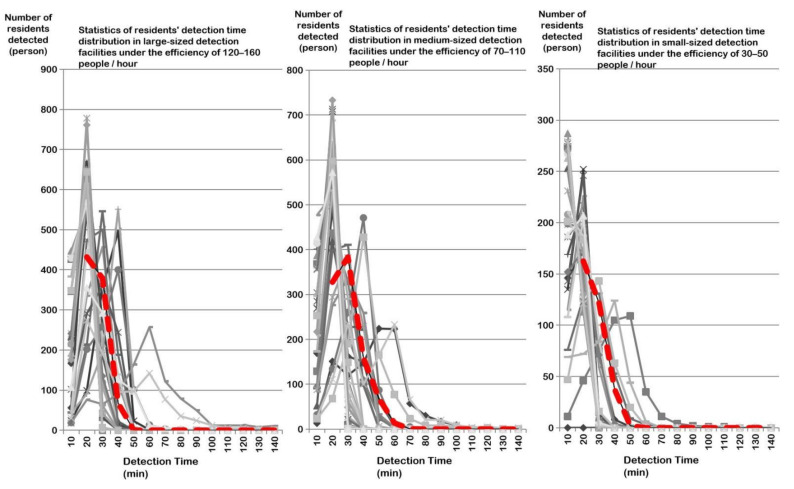
Statistical chart of residents’ time distribution in community primary testing at the efficiency of three levels (30 times for each simulation).

In the late COVID-19 pandemic period, the agent parameters for resident behavior did not require complex settings due to the streamlined testing process. Virus-testing efficiency values for three levels of community health service centers were also obtained after 30 simulations, with large spaces accommodating 120–160 persons/h, medium spaces accommodating 70–110 persons/h, and small spaces accommodating 30–50 persons/h (Figure 8).

### 3.2. Evaluation of Testing Efficiency of Community Health Service Centers in Wuhan Based on “Community Testing Facility Service Efficiency”

Based on the results of the simulation analysis, we evaluated the testing efficiency of community health service centers throughout Wuhan City.

#### 3.2.1. Evaluation of Testing Efficiency in Community Health Service Centers in the Early COVID-19 Pandemic Period

**Figure 9:**
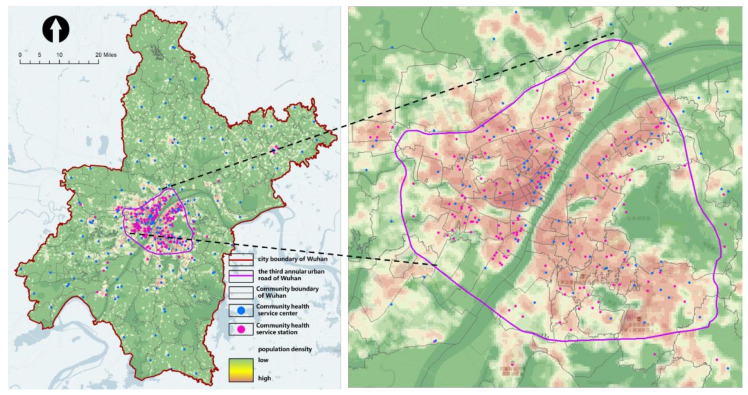
Heat map of the population during the COVID-19 pandemic in Wuhan City.

In the early period of the COVID-19 pandemic, the spatial distribution of residents (heat map) shows that the population density in the main urban area is significantly higher than that in the suburbs, and the population density in Hankou district in the main urban area is higher than that in the Hanyang and Wuchang districts (Figure 9).

In the early period of the COVID-19 pandemic, the medical resources of large hospitals were mainly focused on treating patients, and the testing work was basically undertaken by community health service centers as required. Referring to the information in the “Pneumonia Treatment Protocol for COVID-19 Infection (6th Trial Version)” that “the incubation period of the virus is mostly 3–7 days”, we determined 2 time values of 3 days (36 h) and 7 days (84 h) as the “pass” threshold of time required for the community to complete the testing work (otherwise, it is “fail”) based on the standard of 12 working hours per day. After 7 days, the exceedance time continued to accumulate according to the 7-day (84 h) threshold, in order to evaluate the overall situation of the time taken by the communities in Wuhan to complete virus testing.

**Figure 10:**
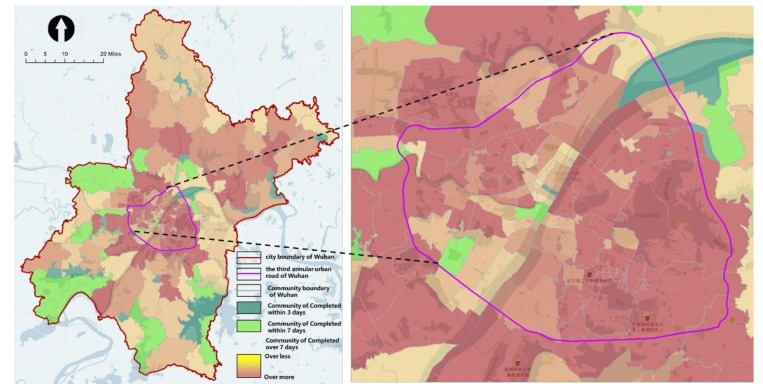
Time taken to complete community virus testing in Wuhan (in the early period of the COVID-19 pandemic).

The assessment results show that the community testing capacity in the main urban area is inadequate. In terms of the proportion of communities meeting the standards, 17 communities (shown in dark green) were able to complete the testing requirements within 3 days, covering only about 1% of the population. The 19 communities (in light green) that could complete the requirements in more than 3 days or less than 7 days, covering about 3% of the population. A sum of 109 communities with mild and moderate non-compliance (in light yellow) covered about 68% of the population. There were 38 communities with severe non-compliance (in dark yellow and red), covering about 49% of the population (Figure 10).

In terms of spatial distribution, the spatial clustering analysis using the “Optimized Hotspot Analysis” tool showed that most of the substandard communities (in dark red) were concentrated in and around the main urban area, especially on the south of the Yangtze River.

#### 3.2.2. Evaluation of Testing Efficiency in Community Health Service Centers in the Late COVID-19 Pandemic Period

**Figure 11:**
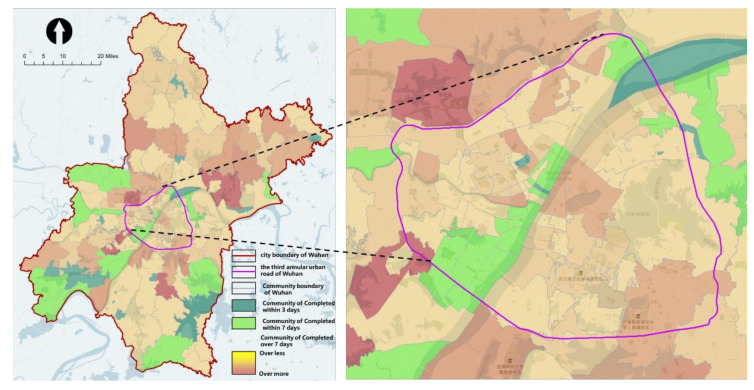
Time taken to complete community virus testing in Wuhan (in the late COVID-19 pandemic period).

**Figure 12:**
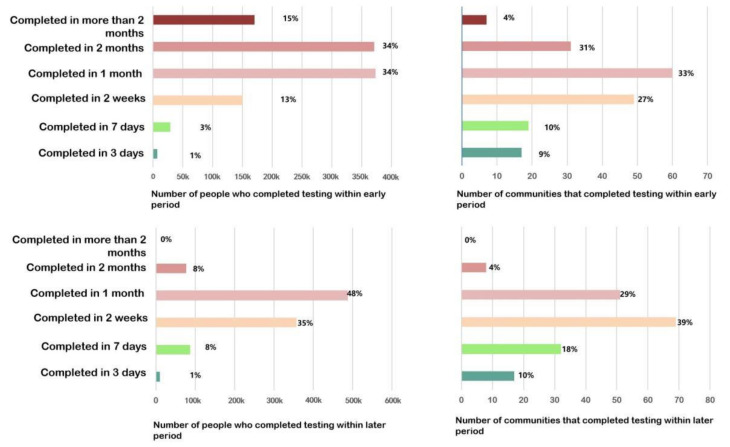
Distribution of ratios of communities completed virus testing and the population covered in Wuhan in different periods.

In the late COVID-19 pandemic period, as their medical resources were no longer strained, large hospitals started to dispatch staff and equipment to carry out virus testing together with community health service centers and community health service stations to improve the efficiency of testing. According to the government’s plan, 61 full-time fever clinics (hospitals of Class-2 Grade-A or higher) were allocated to perform the testing work together. According to the incomplete survey, 40% of the testing population could be served, while the remaining 60% of the population had to be tested at community health service centers. According to the simulation, the results showed that the testing capacity of the community had improved: 28% of the communities could complete the testing within 7 days, accounting for 9% of the residents; 39% of the communities and 35% of the population could complete the testing between 7 and 14 days, respectively. In terms of spatial distribution, spatial clustering analysis using the “optimized hotspot analysis” tool showed that there were no longer obvious hotspots (Figure 11 and Figure 12).

It can be seen that the late period of the COVID-19 pandemic, with the improvement in the detection process and the efficiency, the scale of communities and residents that can complete the detection according to the limit time requirements has been greatly improved, but there is still a gap between the actual state and the idealized goal. Further analysis and exploration is required to achieve the most effective scheme.

## 4. Discussion

Based on the Quant theory, the improved Wells-Riley formula, and some respiratory infectious disease virus parameters, we conducted several simulations in this paper on the optimal virus-testing efficiency in the experimental scenarios within the certain-exposure time in community health service centers in the early and late periods of the COVID-19 pandemic. Furthermore, we evaluated the testing efficiency of community health service centers throughout Wuhan. The following conclusions were drawn.

In the early COVID-19 pandemic period, the reasonable testing service efficiency of community health service centers was about 300 persons/h, which is about 15–20% lower than the maximum load efficiency. In the late COVID-19 pandemic period, the testing service efficiency of large, medium, and small community health service centers was 120–160 persons/h (large), 70–110 persons/h (medium) and 30–50 persons/h (small), respectively. In contrast, the reasonable testing service efficiency obtained from agent-based simulation takes into account both testing exposure time and testing efficiency, as well as various factors such as testing process, testing facility size, testing facility standard, testing population and decision-making behavior ratio. Some scholars have focused on the spread of COVID-19 in both macro-scale and micro-scale spaces by Anylogic. Though there were some similar micro-scale studies [46,47,48], all of these were based on an ideal experimental environment. With a relatively lack of practicality and few number of experimental simulations, the conclusions are of some contingency. The simulation environment of this paper is as close as possible to the actual situation (Wuhan), and the result is verified to be reliable with multiple simulation statistics.

In the early COVID-19 pandemic period, only 4% of communities and 19% of residents in Wuhan were tested within 7 days. In the late COVID-19 pandemic period, about 8% of communities and 28% of residents in Wuhan were tested within 7 days, and 35% of communities and 39% of residents were tested within 2 weeks. Both in the early and late periods of the COVID-19 pandemic, more than half of the residents were at higher risk of infection due to the high density and prolonged clustering of residents during the virus testing. Currently, only one case of cross-infection and widespread transmission during testing was reported in Hangzhou, China, indicating that cross-infection does occur during testing, while no case was reported in Wuhan. On the one hand, all of the communities in Wuhan were organized for virus testing in an orderly manner, in batches and at different times. On the other hand, the government commandeered schools and gymnasiums to set up dozens of temporary testing sites to relieve the pressure on community health service centers.

In the early COVID-19 pandemic period, the large size of most communities south of the Yangtze River and the large population under their jurisdiction significantly affected the testing efficiency of community health service centers, resulting in a higher probability of virus infection (including testing and other scenarios). Although the population density is higher in the area north of the Yangtze River, the population size of individual communities is relatively smaller due to the subdivision of communities, and virus testing can be conducted within the required time. From the study of the service level of community health service centers in Wuhan, it is clear that the service level of community health service centers in the region south of the Yangtze River is indeed limited due to the small number of large-scale community health service centers, the unreasonable spatial distribution, and the mismatch between community size and health center service capacity.

In response to these findings, the following recommendations are made large-scale virus testing that may be needed in the future:

By further investigation and simulation to determine the Quanta parameters of the COVID-19 infection rate (especially for new virus variants such as delta), the scientific nature of the “certain-exposure time” should be strengthened to lay a good foundation for the rational organization of virus testing for a large population;

Community health service centers should be better equipped (with a focus on the main urban areas, especially in communities south of the Yangtze River) to cope with the strained health care resources and complex testing tools during the early COVID-19 pandemic period (and similar respiratory infectious diseases). The testing capacity of health service stations should be expanded to support rapid and full testing in the late COVID-19 pandemic period;

Communities with severely prolonged testing times and excessive population should consider increasing the number of community health service centers and health service stations;

Based on the population density of the community and the testing capacity of community health service centers, the flow of personnel involved in testing should be integrated and controlled to break the constraint of testing only by the administrative boundaries of communities, so that the testing demand and testing capacity can be better matched.

## 5. Conclusions

In this paper, the agent-based simulation model is a complex model, which requires many parameters and multiple scenarios. Since the COVID-19 pandemic outbreak, studies on complex model parameters have been limited, and parameter omissions may lead to biased or limited simulation results. This paper tries to simulate the real operation of community health service facilities as much as possible and provide reference for the practical community virus detection work. In reality, there are many specific conditions, such as changes in the number of people examined, different equipment and space, adjustment of the detection process, etc. Therefore, the more careful the conditions set in this paper, the more convenient for other regions to reference, choose and adjust the conditions in this manuscript combined with their own needs. Thus, the results of this paper have a high degree of overlap with the actual situation and have a strong reference value and significance. On the one hand, the spread of the COVID-19 pandemic in Wuhan City can be prevented and controlled. On the other hand, this research paradigm has a wide application value and can be used as a reference for virus testing and the COVID-19 pandemic prevention and control in other cities.

One of the main limitations of the study is the lack of in-depth understanding of the pathogenic mechanism of COVID-19 infection. Wearing masks, maintaining social isolation distance, increasing ventilation, frequent disinfection and hand-washing may greatly reduce the chance of infection, so that the control standard of detection time is relaxed (120 min upper limit). In addition, the delta variant has a higher chance of infection, and may substantially require the reduction in the detection time control standard. However, once the medical field has new research conclusions, our paper can quickly adjust the model parameters to update the results and recommendations. Anylogic has proved that the simulation study of COVID-19 infection transmission can be assisted from multiple perspectives and levels. This paper can be coupled with some partial medium micro model techniques, such as introducing different number of infectors in agent to analyze whether the equipment layout and shape of different community health service facilities can affect the virus transmission efficiency, in order to provide reference for the planning and design of community health service facilities. Thus, the future research will continue in the following two aspects.

Firstly, through the deepening understanding of COVID-19 and further updating of the simulation model, more accurate operational prerequisites and realistic simulations can be extracted to contribute more to the new round of efficient epidemic prevention and control. Secondly, it can improve the accurate evaluation of the testing capacity of community health service centers. By maturing the pattern and upgrading the model, we can make a more comprehensive, detailed, and practical evaluation of community health service centers, especially those located outside the main urban area.

## Figures and Tables

**Table 1:** Data types and acquisition methods during the COVID-19 pandemic.

Data Type	Data Description	Data Source	Collection Method
Demographic data	Total population size of Wuhan City	Baidu Digital Intelligent Platform Background	Provided by the cooperation platform
	Community population size		
Community data	Community boundaries and spatial distribution	Published by Wuhan Municipal Government	Provided by the cooperative platform
Data from community health service centers	Scale and spatial distribution of community health service centers	List published by the Wuhan public data development platform	Collected by official website
	Scale and spatial distribution of community health service stations		
	Testing process, medical staff and equipment configuration	Government-set standards	Collection on official websiteField survey
Proportion of tested residents data	Size and proportion of population for initial temperature measurement, second temperature measurement, throat swab and blood test, CT scan	Sina Weibo Official press release	Web search
Residents’ behavioral characteristics during testing	Self-determination of whether to conduct virus testing	Voting on Sina Weibo	Data collection on Weibo website

**Table 2:** Transformation of testing process and simulation setup in community health service centers.

Period	Early Period			Late Period				
	**Procedure**	**No. of Windows**	**Proportion**	**Procedure**	**Facility Scale**			**Proportion**
					**Large**	**Medium**	**Small**	
Test Content	First temperature measurement	4	41.53%	None	--			--
	Second temperature measurement	6	10%	None	--			--
	Register, get test tubes and do throat swabs	3	23.12%	Register, get test tubes and do throat swabs	4	3	2	100%
	Blood drawing	3		Blood drawing	3	2	1	
	CT	1		None	--			--
	Doctor consultation	3		None	--			--
Checked Residents	10.58 million × 41.53% × 23.12%			10.58 million × 60%				

## Data Availability

Data sharing not applicable.
